# The Attitude of Great Writers Towards Disease

**Published:** 1892-12-10

**Authors:** 


### The Attitude of Great Writers Towards Disease.

### XVIII.—MILTON.

If anywhere it might be thought some deep philosophy of pain would be discoverable in the work which set out to "justify the ways of God to man." must not the whole idea of a lost paradise, the tracing of the flood of human misery from its far off source set the poet upon an attempt to fathom the mystery implied in a world of pain? In asserting eternal providence must he not shape some theory to account for its decrees on earth?

It is remarkable that Milton seems to have formed no such theory nor made any such attempt. He is not farther from any endeavour to build up the six days' creation on a scientific basis, than he is from any divergence from the sternly dogmatic and high-handed theology current in his day. Here is no neat scheme of the universe warranted to answer all objectors, nor any restless sounding of unfathomable problems. Of such he wrote:—

> They ravel more, still less resolved,
> But never find self-satisfying solution.

But if he refuses to theorise about the cause of suffering, Milton lent his hightest endeavours to painting its effects. The righteous decrees of God suffice him for its infliction; but how man may best adapt himself, best endure, profit, and "overcome by suffering," he had the best of all reasons for understanding.

The simplest view of disease is, that it is the direct consequence of sin. This is how it appeared to Adam when Michael would show:

> What misery the inabstinence of Eve
> Shall bring on men. Immediately a place
> Before his eyes appeared, sad, noisome, dark;
> A lazar-house it seemed; wherein were laid
> Numbers of all diseased; all maladies
> Of ghastly spasm, or racking torture, qualms
> Of heart-sick agony, all fevrous kinds,
> Convulsions, epilepsies, fierce catarrhs,
> Intestine stone and ulcer, colic pangs,
> Demoniac phrensy, moping melancholy,
> And moonstruck madness, pining atrophy,
> Marasmus and wide wasting pestilence,
> Dropsies and asthmas, and joint racking rheums
> Dire was the tossing, deep the groans; despair
> Tended the sick, busiest from couch to couch;
> And over them triumphant death his dart
> Shook, but delayed to strike, though oft invoked
> With vows, as their chief good and final hope.

One may wonder if this picture were drawn by Milton from some actual knowledge of the interior of a hospital in his day. At any rate, here is pain in its cruellest light, as punishment pure and simple, without alleviation or purifying influence. As such in hardly varied decree it was assigned to Satan and his angels at their fall, though Milton's infernal region, with its varied pursuits and harmonious sadness, knows no such scenes as those above quoted. The real hero of the epic is nowhere more magnificent than in his first encounter with irrevocable pain; whether he proclaims that

> To be weak is miserable,
> Doing or suffering, or surveying his new estate, speaks the words so characteristic of Milton himself:

> The mind is its own place, and in itself
> Can make a heaven of hell, a hell of heaven.

To a point somewhat higher than this stoical endurance of righteously-incurred suffering Samson finally attains, but he is first seen in the throes of an anguish too deep and present to admit of philosophic consolations.

> Oh! dark, dark, dark, amid the blaze of noon,
> Irrevocably dark, total eclipse,
> Without all hope of day.

The passage holds deep pathos if we may believe it wrought out of the bitter personal experience of the poet.

> My griefs not only pain me,
> As a lingering disease,
> But finding no redress, ferment and rage,
> Not less than wounds immedicable
> Rankle, and fester, and gangrene,
> To black mortification.
> Thoughts, my tormenters, armed with deadly stings.
> Mangle my apprehensive tenderest parts,
> Exasperate, exulcerate, and raise
> Dire inflammation, which no cooling herb
> Or medicinal liquor can assuage,
> Nor breath of vernal air from snowy Alps.
> Sleep hath forsook and given me o'er
> To death's benumbing opium as my only cure.

So far we have seen disease revealed to Adam by Michael as a terrible dispensation of punishment, serving sorely to awaken terror and remorse; next, the purely stoical yet wholly noble attitude of Satan in face of irremediable pain; then the effect of disease on man exemplified by Samson; his rise out of despair under the influence of

> Some source of consolation from above, till he attains an atmosphere of patient endurance equal in degree to that of Satan, but how widely differing in kind!

Yet this patience is unsustained by hope, unless it is the hope of a speedy ending to life and suffering both. The patience which hopes must be taught by the great Master of suffering, and it is to Him Milton ascribes the words which set pain again in a higher aspect, purifying, preparing, and educating the human soul for a purpose not yet revealed. It may read almost as an anwer to Satan's query, "Lives there who loves his pain?"

> What if He hath decreed that I shall first
> Be tried in humble state, and things adverse,
> Suffering, abstaining, quietly expecting,
> Without distrust or doubt, that He may know
> What I can suffer, how obey? Who best
> Can suffer, best can do; best reign, who first
> Well hath obeyed.

But in his personal experience of blindness and poverty barring him from the work in his country's service which he held most sacred, Milton advances yet a step farther, and in words, which to countless sufferers since have been a stay of comfort, proclaims suffering to be not merely a preparation for work, but work itself, and bids the weak and helpless remember that God's eternal purposes are wrought not more surely by the strong enthusiast than by those whose daily task is no greater than to bear for Him the day's burden of pain.

> God doth not need
> Either man's work, or His own gifts; who best
> Bear His mild yoke, they serve Him best: His state
> Is kingly; thousands at His bidding speed,
> And post o'er land and ocean without rest;
> They also serve who only stand and wait.

